# Editorial: Emerging trends in global women's health—sex and gender differences in disease

**DOI:** 10.3389/fgwh.2023.1304382

**Published:** 2023-10-23

**Authors:** Irene Göttgens, Sabine Oertelt-Prigione

**Affiliations:** ^1^ Department of Primary and Community Care, Radboud University Medical Center, Nijmegen, Netherlands; ^2^ AG 10 Sex- and Gender-Sensitive Medicine, Medical Faculty OWL, University of Bielefeld, Bielefeld, Germany

**Keywords:** women’s health, sex categorization, gender dimensions, trends, equity in health care

**Editorial on the Research Topic** Emerging trends in global women’s health—sex and gender differences in disease

Over the years, the discussion surrounding the implications of sex characteristics and gender dimensions on health outcomes has shifted from a fringe topic to a central concern in scientific research. Global health trends and the COVID-19 pandemic have underlined the significance of understanding the differential impacts of diseases across genders. This Research Topic brings together a collection of papers exploring the various dimensions of women's health, highlighting sex and gender disparities in disease manifestation and outcomes.

In their brief investigation, Ferretti et al. tap into the realm of behavioural patterns by exploring the evolving trends of smoking prevalence in women across 191 countries from 1990 to 2019. Their finding that smoking habits are not universally convergent but cluster in specific “clubs” provides a lens to understand regional variations in smoking habits of women. Intriguingly, the role of economic factors, especially cigarette affordability, emerges as a significant determinant of these smoking trends, shedding light on the interconnectedness of socioeconomic policies and smoking behaviours among women.

The enduring challenge of Human Immunodeficiency Virus type-1 (HIV-1), especially its disproportionate impact on women, is brought into focus by Rao. Their mini-review sheds light on the urgency of understanding how biological sex characteristics, such as an XX-sex chromosomal karyotype and menopausal transition, influence viral reservoir characteristics and immune responses in cis-women. They report that sex-specific differences in HIV-1 may hinder the development of a scalable cure for women living with HIV-1 (WLHIV). The authors call for comprehensive HIV-1 cure research that ensures sufficient representation of WLHIV and the systematic inclusion of sex stratification in data reporting of drug responses and viral reservoir locations and consideration of sex differences in clinical trial endpoints.

Hallam et al. provide an insightful perspective on the trajectory of sex and gender research (SGR) in health and draws attention towards skill development of early- and middle-career researchers (EMCRs). Their recommendation includes three pivotal dimensions that can potentially improve future SGR practices: synergizing the objectives of SGR with the responsibility of addressing systemic biases, expanding SGR to include intersectional approaches and elevate the importance of “null findings”. The authors underscore that concerted efforts on these avenues can indeed pave the way for tangible advancements in reducing health disparities on a global scale.

In a brief research report, Buslón et al. bring attention to the intersections of technology and sex- and gender related biases, notably in the burgeoning field of Artificial Intelligence (AI) in health. Their concerted effort to spotlight sex and gender biases in AI research echoes the global call to champion diversity and inclusivity in technological advancements. In their brief report, they offer key recommendations from the Bioinfo4women (B4W) program to facilitate the inclusion of sex and gender perspectives in AI and health research.

Lastly, the ramifications of the COVID-19 pandemic on gender dynamics form the focus of the original study by Hoffmann et al. Their qualitative exploration underscores the unique challenges faced by highly educated women working in global health in different European counties during the pandemic, from amplified domestic responsibilities to shifts in professional demands. The silver lining, perhaps, is the potential of women-centric networks like Women in Global Health (WGH) in fortifying resilience and fostering collaboration during crises.

Taken together, these papers provide a panoramic view of different initiatives addressing global women's health and illuminating the multi-faceted challenges and opportunities that lie ahead. It is our hope that this Research Topic sparks renewed interest and ignites collaborative efforts in addressing the health inequities that persist across genders. The journey towards a more equitable healthcare landscape requires persistent inquiry, and these papers offer knowledgeable examples for the incorporation of sex and gender considerations into global health research. We anticipate that health researchers, regardless of their niche, will glean actionable insights for their own work from these contributions.

